# Maternal Anthropometric Factors and Circulating Adipokines as Predictors of Birth Weight and Length

**DOI:** 10.3390/ijerph17134799

**Published:** 2020-07-03

**Authors:** Dominika Mazurek, Monika Bronkowska

**Affiliations:** Department of Human Nutrition, Faculty of Biotechnology and Food Science, Wrocław University of Environmental and Life Sciences, ul. Chełmońskiego 37, 51-630 Wrocław, Poland; monika.bronkowska@upwr.edu.pl

**Keywords:** adiponectin, leptin, weight gain, BMI, birth weight, birth length

## Abstract

Pregnancy is a period of serial metabolic and hormonal changes in the woman’s body. Factors such as circulating adipokines affect the fetal period and may cause long-term changes in metabolic pathways at the cellular, tissue, or organ level. The nutritional status of the pregnant woman affects the course of pregnancy, delivery, and confinement, as well as the health of the offspring following birth and in subsequent years. Adipokine hormones essential for modulating metabolism during pregnancy include adiponectin and leptin. This study aimed to assess maternal anthropometric parameters and plasma concentrations of specific adipokines as predictive measures of newborn birth weight, birth length, and ponderal index. Anthropometric measurements (prepregnancy body weight and height) were obtained from 168 surveyed Polish women. Data related to the birth parameters of 168 newborns (body length and mass) were derived from clinical records. Circulating maternal adiponectin and leptin levels at birth were determined. Significant correlations between newborn birth weight and maternal prepregnancy body mass index (*p* < 0.05) or maternal weight gain during pregnancy (*p* < 0.05) were observed. Women with below normal weight gain during pregnancy were more likely to give birth to newborns with significantly lower birth weight than women with excessive weight gain during pregnancy (*p* < 0.05). Maternal plasma concentrations of leptin were significantly related to prepregnancy maternal body mass index (*p* < 0.05), and concentrations of adiponectin and leptin were significantly related to weight gain during pregnancy (*p* < 0.05). However, they did not affect the birth parameters of the newborn.

## 1. Introduction

Body mass index before pregnancy and weight gain during pregnancy are parameters dependent on the woman’s lifestyle and affect the course of pregnancy, fetal development, and newborn health [1,2,3]. Overweight and obesity in women before and during pregnancy are one of the most important risk factors for maternal and fetal complications. The body mass index (BMI) is the indicator commonly used to diagnose and assess the degree of obesity. Elevated BMI values in prepregnancy increase the risk of gestational diabetes, gestational hypertension, and thromboembolism during pregnancy. High BMI values increase the risk of premature births and caesarean sections. Women with excessive body mass before pregnancy more often give birth to children with macrosomia and perinatal injuries [3,4,5]. Nonetheless, weight gain during pregnancy is important for healthy fetal development. Excessive weight gain during pregnancy increases the risk of pregnancy-induced hypertension, gestational diabetes, pre-eclampsia, miscarriage, and perinatal complications (premature birth, fetal death, and fetal deformity) [6,7,8]. Among mothers with excessive body weight, there is a higher risk of fetus classified as large for gestational age (LGA), or small for gestational age (SGA), and newborns with macrosomia (>4000 g at birth) [9]. Low weight gain during pregnancy increases the risk of fetal growth restriction, miscarriage, premature delivery, stillbirth, or perinatal death of the newborn. Underweight women have a lower placenta weight, which in turn leads to reduced placental transport of fetal nutrients [2,10,11].

Adiponectin is a tissue hormone protein with trophic effects. It is synthesized and secreted mainly by cells of the adipose tissue (adipocytes). Adiponectin has anti-inflammatory and antiatherosclerotic effects and regulates appetite and insulin sensitivity of cells. The concentration of adiponectin is negatively correlated with body fat [12,13,14,15]. During pregnancy, the concentration of adiponectin decreases due to an increase in the amount of adipose tissue in the female body, while its concentration increases in umbilical cord blood plasma. According to Teler et al., this increase is related to the synthesis of adiponectin in placental syncytiotrophoblasts and is regulated by IFN, IL-6, TNFα, and leptin [16]. The adiponectin concentration is significantly lower in obese pregnant women compared to women with normal body weight, and is inversely correlated with the birth weight of newborns [16]. Low levels of maternal adiponectin may reduce placental amino acid transport and inhibit fetal growth [17,18]. During pregnancy, the concentration of adiponectin is important for the development of gestational diabetes, because it regulates the sensitivity of the body’s cells to insulin and glucose homeostasis. A low concentration of this adipokine in the blood plasma of a pregnant woman is positively correlated with a higher incidence of insulin resistance, gestational diabetes, and low birth weight [19,20,21].

Leptin is a protein with tissue hormone properties, secreted mainly by adipocytes of white adipose tissue. Its synthesis is induced by the expression of the obesity gene (*ob*). Leptin regulates energy, immunological, and reproductive processes. It participates in angiogenesis and the metabolism of carbohydrates (mainly glucose and glycogen) and fats (lipid oxidation). The concentration of leptin in the body is closely related to the volume of adipose tissue [14,16,22,23]. During pregnancy, there is an increased level of leptin in the mother’s body due to increases in total fat content, synthesis of this adipokine in the human placenta, and increasing energy needs of the mother, placenta, and fetus [16,24,25]. According to Markowska et al., the most significant increase in leptin concentration occurs in the first trimester of pregnancy; from the second trimester it remains at a constant, high level [22]. Leptin is involved in optimizing the availability of substrates necessary for fetal growth, particularly by mobilizing maternal fat stores that are significantly correlated with the birth weight of the newborn. According to Perichart-Perera et al. (2017), pregnancy leptin concentration could be a predictor of infant size at birth and is related to mother’s weight [9]. Leptin plays a crucial role in embryo implantation, induces the synthesis of human chorionic gonadotropin (hCG), regulates mitogenesis, and stimulates placental development. It is likely that this adipokine regulates fetal haemopoietic cell proliferation and lung development and stimulates surfactant synthesis [16,20,25].

The first significant period in a person’s life is the intrauterine period. Stimulants that affect the fetal period may cause long-term changes in the metabolic pathways of the body (at the cellular, tissue or organ level). The lifestyle of the woman and her body’s state of nourishment (concentration of selected modulators of metabolism and the degree of body fat) affect the course of pregnancy, delivery, puerperium, and the health of the children after birth and in subsequent years of his or her life [26]. The factors mentioned above defined the most important goal of the study: to assess the concentration of selected adipokines in the mother’s blood and the mother’s anthropometric parameters as factors determining the birth weight and length of the newborn.

## 2. Materials and Methods

### 2.1. Study Group

The study was conducted among 168 women from Wroclaw, Poland in the years 2015–2017. The study group included women in the third trimester of pregnancy, admitted to the obstetric-gynecological ward of the Wroclaw Medical University, for delivery by caesarean section. The study inclusion criteria were as follows: age between 18 and 40 years, singleton, uncomplicated pregnancies (without gestational hypertension, pre-eclampsia, gestational diabetes), declaration of good health condition (declared by an obstetrician), and delivery by caesarean section. The use of caesarean section was due to the patient’s demand, caesarean section in the past or at the suggestion of another specialist such as an ophthalmologist (e.g., retinal detachment) or orthopedist (e.g., abnormal structure of the pelvis). Among the subjects, two age groups were distinguished: <30 and ≥30 years of age. All women gave their informed consent for inclusion before they participated in the study. The study was conducted following the Declaration of Helsinki, and the protocol was approved by the Ethics Committee of Wroclaw Medical University (KB-85/2015).

### 2.2. Anthropometric Measurements

Anthropometric measurements, i.e., body weight before pregnancy, body weight before delivery, and height, were obtained from the 168 women surveyed. The body weight before birth and height were derived from clinical records. The body weight before pregnancy was given by the patient. Based on the anthropometric data obtained, the BMI before pregnancy and weight gain during pregnancy were calculated and evaluated according to the criteria below.

BMI was calculated from the following formula: BMI (kg/m^2^) = body weight (kg)/body height (m)^2^. Maternal prepregnancy BMI was categorized into clinical categories according to World Health Organization (WHO) cutoffs: underweight < 18.5 (kg/m^2^), normal weight 18.5–24.9 (kg/m^2^), overweight 25.0–29.9 (kg/m^2^), obesity ≥30.0 (kg/m^2^) [27].

Data on gestational weight gain (kg), defined as the difference between the latest weight before delivery and prepregnancy weight, were categorized in relation to maternal prepregnancy BMI according to the guidelines of the US Institute of Medicine (IOM) (Table 1) [28,29].

Birth weight and length of 168 newborns were derived from clinical records. The birth weight of newborns was assessed according to the following criteria [10]: <2500 g, low birth weight; 2500–3999 g, normal birth weight; and ≥4000 g, high birth weight. Birth length was assessed according to the following criteria [30]: <46 cm, short birth length; 46–54 cm, normal birth length; and >54 cm, long birth length.

The Ponderal index (PI) was calculated based on birth weight and birth length. The PI was calculated using the following formula [31,32]: PI (g/cm^3^) = (birth weight (g) × 100)/birth length (cm)^3^. The values obtained were interpreted according to the following criteria [33]: <2.0 g/cm^3^, below standard values; 2.0–2.5 g/cm^3^, below standard values but acceptable; 2.5–3.0 g/cm^3^, within standard values; and >3.0 g/cm^3^, above standard values.

### 2.3. Biological Material

Venous blood was collected on the day of admission to the maternity ward before giving medicines (after 39 weeks of pregnancy). Plasma was isolated from blood samples and stored at −80 °C for downstream analysis. Biochemical markers in maternal plasma were determined in the laboratory of the Department of Human Nutrition at the University of Life Sciences in Wroclaw. Quantification of markers was determined using ELISA enzyme immunoassays. In the mother’s blood plasma, adiponectin and leptin were determined. The concentration of adiponectin in maternal plasma was determined using a self-coating Human Adiponectin/Acrp30 DuoSet ELISA kit (catalogue no. DY1065; R&D Systems, Bio-Techne, Minneapolis, MN, USA). In order to determine the concentration of adiponectin, dilution of plasma was performed at 1:30,000 according to the manufacturer’s guidelines. The concentration of leptin in the maternal plasma was determined using a self-coating Human Leptin DuoSet ELISA kit (catalogue no. DY398-05; R&D Systems, Bio-Techne). In order to determine the concentration of leptin, plasma was diluted at 1:100 following the instructions provided with the test. The readings were done on an Epoch microplate spectrophotometer from BioTek Instruments.

### 2.4. Statistics

Statistical evaluation of the results was carried out using the StatSoft STATISTICA 12.5 PL (StataCorp LP., College Station, TX, USA) computer program. In order to check the normality of distribution of continuous features, the Kolmogorov–Smirnov test with the Lilliefors correction was used. Most of the test results evaluated did not show normal distribution, except for the PI. To test for statistically significant differences between groups, we used: Student’s *t*-test and Tukey’s ANOVA test for different N for variables where normal distribution was shown; and Mann–Whitney U test and Kruskal–Wallis test for variables where normal distribution was not shown. In this work, the results for data with normal distribution were expressed as mean and standard deviation. In contrast, data without normal distribution were presented as median and quartiles. Multiregression models (with logarithmic transformation where normal distribution was not shown) were performed using the ponderal index, weight, and length of the newborn as dependent variables; adiponectin and leptin concentration, prepregnancy BMI, and gestational weight gain of the mother were considered as independent variables. Next, multiregression models were performed using adiponectin and leptin as dependent variables, and prepregnancy BMI, gestational weight gain, and age of mother as independent variables. Statistical significance was considered at *p* < 0.05.

## 3. Results

### 3.1. Anthropometric Parameters of the Mother

We evaluated the relationship between anthropometric parameters of the women examined before and during pregnancy and the birth parameters of the newborns. Table 2 shows the characteristics of the studied women; Table 3 shows the characteristics of the studied neonates.

**Figure 1:**
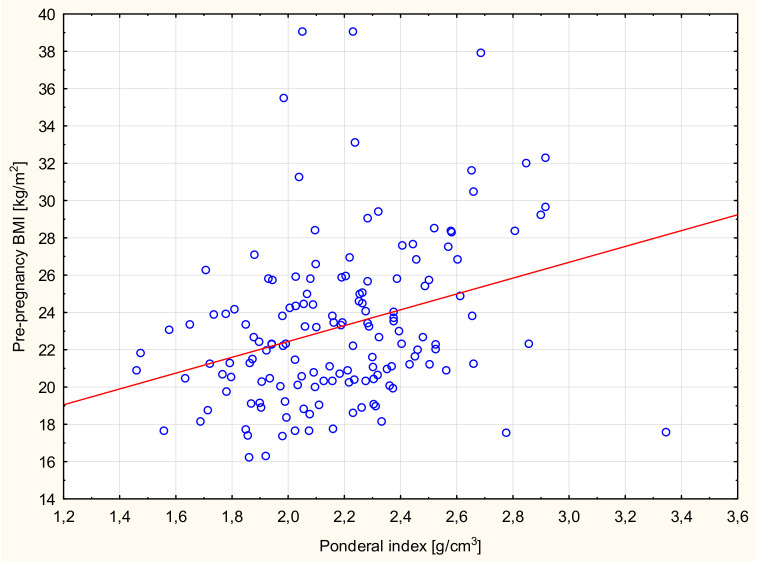
Scattergram of neonatal Ponderal index (PI) dependence and prepregnancy body mass index (BMI) of the examined women, Spearman’s correlation *r* = 0.32; *p* < 0.0001.

**Figure 2:**
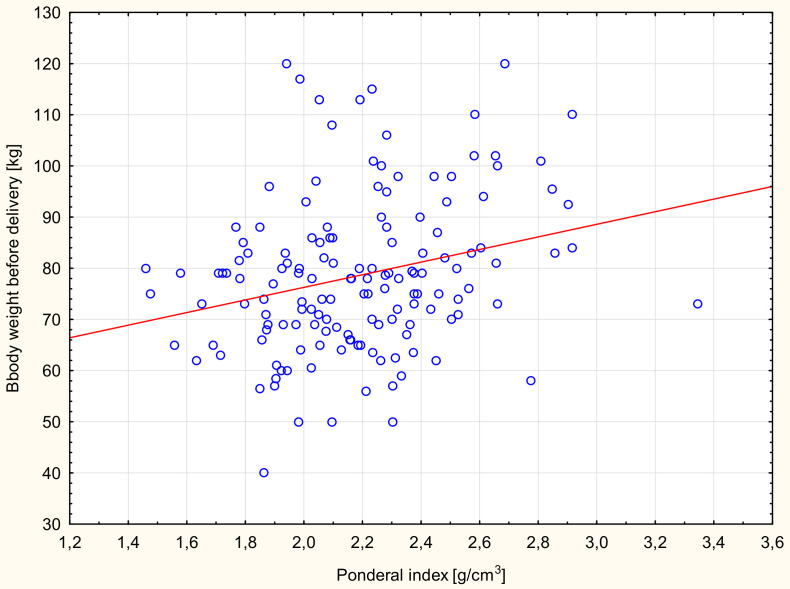
Scattergram of neonatal PI dependence and body weight before delivery of the examined women, Spearman correlation *r* = 0.25; *p* = 0.002.

**Figure 3:**
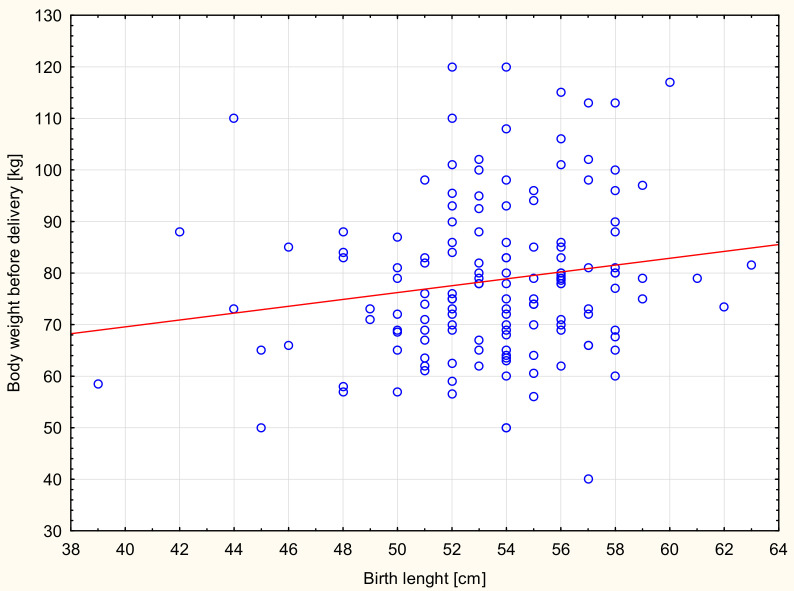
Scattergram of neonatal birth length dependence and body weight before delivery of the examined women, Spearman correlation *r* = 0.17; *p* = 0.03.

Significant correlations were found between the birth weight values of newborns and prepregnancy BMI (Table 4). A similar relationship was found between the birth weight of newborns and women’s weight gain during pregnancy. Women with weight gain in pregnancy below the norm gave birth to newborns with a significantly lower weight than women with an increase in body weight above the norm (Table 5). Significant correlations also were found between the PI values of newborns and prepregnancy BMI (Figure 1) and body weight before delivery of the examined women (Figure 2). The study showed statistically significant correlations between the birth length of newborns and body weight before delivery of the studied women (Figure 3).

### 3.2. Modulators of Metabolism

The concentration of the selected adipokines, adiponectin and leptin, was determined in the blood plasma of the pregnant women. The median adiponectin concentration in the blood plasma of the pregnant women was influenced by age (Table 6). Women ≥30 had significantly higher concentrations of adiponectin in blood plasma than women <30 years of age. It was also found that adiponectin concentration was inversely proportional to body weight during pregnancy. The associations between mother adiponectin concentrations and offspring anthropometry at birth were not significant.

The analyses showed significant differences between the concentration of leptin in the blood plasma of the studied women and their anthropometric indicators (Table 7). It was found that leptin concentration in plasma was directly proportional to the BMI and weight gain during pregnancy. There were no statistically significant relationships between plasma leptin concentration and offspring anthropometry at birth.

The analysis of data with a multiple regression model revealed that the adiponectin concentration was negatively associated with weight gain during pregnancy and that the leptin concentration was positively associated with prepregnancy BMI and weight gain during pregnancy (Table 8). Thus, maternal body mass index (BMI) can influence the weight of the newborn and the ponderal index. This second indicator could also be predicted by the concentration of leptin (Table 9).

## 4. Discussion

It was shown that the BMI of women before pregnancy and their weight gain in pregnancy had a significant impact on birth weight and PI of newborns, but had no effect on birth length. Women who were overweight or obese before pregnancy gave birth to newborns with higher birth weight and higher PI significantly more often than underweight women or those with a normal body mass. It was also found that women with excessive weight gain during pregnancy gave birth to children with a significantly higher birth weight more often than women with an increase in body weight below the norm.

In the study of Przybyłowicz et al., conducted among 510 women from Olsztyn and Warmia-Masuria, it was shown that weight gain during pregnancy had a significant impact on the birth parameters of newborns (birth weight, length, and PI). Women with a deficient weight gain during pregnancy gave birth to newborns with a significantly lower mass (2750.1 g) or birth length (52.7 cm) than women with a normal (3477.5 g and 55.5 cm) or excessive (3666.4 g and 55.5 cm) body weight gain. A similar trend was observed for the PI of newborns (weight gain: below the norm—PI 19.9 kg/m^3^; standard—PI 20.6 kg/m^3^; above the norm—PI 21.7 kg/m^3^) [6]. In this study, a similar relationship was found: birth weight and PI of newborns were dependent on women’s weight gain during pregnancy. Gala et al. conducted a study among 200 pregnant women from Mumbai who were divided into two age groups: ≤ 27 and ≥ 28 years of age. Anthropometric parameters of mothers and birth rates of newborns were not significantly different depending on the age of the subjects. The authors showed that birth weight and PI of newborns were positively correlated with body weight before and during pregnancy [34]. In a study conducted by Frederick et al. among pregnant women from the USA, the influence of BMI before pregnancy and weight gain during pregnancy on the birth weight of newborns was evaluated [10]. Similar to our results, an increase in BMI before pregnancy and the weight gain of pregnant women were significantly related to an increase in the birth weight of newborns. The authors also emphasized the fact that both deficient and excessive body weight in pregnant women may predispose to too low or too high birth weight of newborns, which in turn may cause numerous nutritional complications in future life [10]. Voerman et al. [35] conducted a data meta-analysis among 162,129 mothers and their children from 37 cohorts from Europe, North America, and Australia. They showed that maternal underweight was associated with lower risks of overweight or obesity throughout childhood and maternal overweight and obesity were associated with higher risks of overweight or obesity throughout childhood. Higher maternal gestational weight gain was associated with higher risk of overweight or obesity in childhood. They suggested that intervention measures to reduce the incidence of overweight or obesity among children should focus on the nutritional status and body weight of the mother before pregnancy, as well as weight gain during pregnancy [35]. Goldstein et al. reported ethnic differences in maternal BMI and gestational weight gain and their impact on neonatal outcomes [36]. They showed that women from the USA and Europe have higher prepregnancy BMI than those from Asia. In the USA and Europe, gestational weight gain above guidelines appeared more often than in Asia, and gestational weight gain below guidelines occurred more often in Asia. They showed that insufficient gestational weight gain was associated with increased risk of low gestational age and preterm birth whereas excess gestational weight gain with increased risk of large for gestational age, macrosomia, and caesarean section [36].

During pregnancy, the body fat content increases, and thus the concentration of adipokines in the mother’s blood changes. The concentration of adiponectin during pregnancy gradually decreases, due to the increase in adipose tissue and increasing concentration of leptin in the blood plasma. Adiponectin controls glucose uptake through the placenta by activating AMP protein kinase and translocating glucose transporters. Thus, higher concentrations of adiponectin may result in increased placental glucose uptake and contribute to fetal fat deposition [37,38]. In this study, the plasma concentrations of adiponectin were significantly different due to maternal age and gestational weight gain. With an increase in body weight gain during pregnancy, the concentration of plasma adiponectin concentrations of the subjects decreased. There was no significant effect of the concentration of this adipokine on neonatal birth parameters. In a study by Eriksson et al. conducted among 30-year-old women at 35 weeks of gestation (*n* = 23) living in Sweden, the mean adiponectin concentration was found to be 9.1 μg/mL (9100 ng/mL) [39]. In a study by Naghshineh et al. conducted among 100 pregnant women from Iran, adiponectin levels of 7.22 μg/mL (7220 ng/mL) were demonstrated [40]. However, adipokine concentrations in pregnant women in this study were significantly lower than in these previous studies. Nanda et al. in their study among 300 pregnant women in the United Kingdom determined the adiponectin concentration in women’s serum and evaluated fetal development and birth parameters of newborns [41]. Similar to our study, they did not observe significant differences in the concentration of this adipokine due to the birth weight of newborns. However, Vernini et al., in a study among 72 mother–newborn pairs, showed that adiponectin concentrations in mothers were negatively correlated with gestational BMI, but positively correlated with birth weight. According to the authors, adiponectin concentration is lower among overweight and obese women [42].

The concentration of leptin gradually increases during pregnancy due to the increase in adipose tissue and synthesis of this adipokine by the placenta. Leptin concentrations increased significantly with an increase in BMI and body weight gain during pregnancy. There was no significant effect of the concentration of this adipokine on neonatal birth parameters. In a study by Vahamiko et al. among 103 pregnant women in Finland, there was a significantly higher concentration of leptin in the plasma of women with a prepregnancy BMI indicating overweight or obesity (45.27 ng/mL) than among women with a normal BMI (31.84 ng/mL). In this study, mean leptin concentrations among subjects were lower compared to the results of both of those sets of authors. However, comparable values for leptin concentration were found in the serum of subjects who were overweight (35.5 ng/mL) or obese (47.4 ng/mL) before pregnancy, as in the study of Vahamiko et al. (with normal body weight—31.84 ng/mL; overweight or obese—45.27 ng/mL) [43]. Differences in adiponectin and leptin concentrations in pregnant women’s plasma in our study and those by other authors may result from ethnic differences and from the time blood samples were taken. The metabolism of a woman during pregnancy is constantly changing, and the time of blood collection is crucial [44]. In a study by Huras et al. among 80 pregnant women living in and around Kraków, significantly higher leptin concentrations were found in the plasma of pregnant women with prepregnancy BMI, indicating overweight or obesity in comparison to women with normal BMI; these results are similar to those obtained in our work [45]. In a study by Samano et al. carried out among 168 pregnant girls from Mexico, the relationship between maternal blood leptin concentration (mean 20 ng/mL) and anthropometric parameters as well as birth anthropometry of children were analyzed. Positive correlations were demonstrated between the concentration of this adipokine and BMI before pregnancy and weight gain during pregnancy, similar to this study. A positive correlation was also found between the maternal leptin concentration and birth weight and length of newborns [46]. Hinkle et al. found positive correlations between maternal adipokines (adiponectin and leptin) with neonatal length and skinfold thickness. Adiponectin was inversely associated with the neonatal length and skinfold thickness among women with obesity. Leptin was positively associated with the neonatal length and skinfold thickness among women with obesity and inversely among women without obesity. Maternal adipokines were associated with neonatal size including length and adiposity, which differed depending on maternal obesity [47]. In our study, there were no similar relationships between the concentration of leptin in pregnant women’s blood plasma and the birth length of the newborn. In a cross-sectional study by Solis-Paredes et al. among pregnant women from Mexico who delivered by cesarean section, the concentration of serum leptin was positively, and the concentration of adiponectin was inversely associated with pregestational BMI and gestational weight gain, as in our study. Leptin is a hormone that regulates energy homeostasis and body weight, and excessive weight gain is related to excessive fat accumulation. Higher levels of adipokines during pregnancy could be explained by an important role of endocrine signals for placental development and fetal growth, and their participation in gluconeogenesis regulation and energy balance in later life. Higher leptin concentrations and excessive maternal adiposity may influence placental functions, for example, the transportation and metabolism of lipids. Excessive transport of lipids to the fetus may impact macrosomia prevalence among newborns [48].

## 5. Strengths and Limitations

This study evaluated the concentrations of adiponectin and leptin in the blood of pregnant women defined by their nutritional status and the birth parameters of the newborn. These tests do not belong to standard biochemical tests performed during pregnancy, while these adipokines have a significant impact on the nutritional status of the mother and the child. The strength of this study was that during blood collection and adipokine determination, we used a well-defined and transparent protocol for sampling, storing. and specifying samples to minimize the occurrence of errors. Blood samples were collected by trained personnel. Medical records were used to collect perinatal information, such as birth weight and length, to improve data reliability.

There are several potential limitations to the study. Firstly, the study group was small, and our results may not be able to be generalized to the population. Moreover, the study sample was mostly characterized by a higher education level and place of residence in the city. Secondly, body weight before pregnancy is given by the patient and can be fraught with error. Body weight before pregnancy should be obtained by measurement by a qualified person and in the set week of pregnancy for each of the women. It would also be important to divide the studied women into more homogeneous groups in terms of body weight. Thirdly, this study only focused on two adipokines; more extensive research (e.g., some more clinical data regarding mothers and newborns) is needed in the future. There could be analyzed other routine hematological and biochemical blood parameters and biochemical urine parameters might include: cholesterol, glucose, triglycerides, FFA, insulin, and insulin resistance. Given that the adipokines adiponectin and leptin are linked to maternal nutritional status, it could be interesting to evaluate the dietary habits of the mothers during the pregnancy. It would be interesting to evaluate the association of the investigated inflammatory-related adipokines (adiponectin and leptin) to other inflammatory parameters, such as IL-6, TNF-alpha, and C-Reactive Protein. A comparison of maternal adipokines and umbilical cord blood adipokines might be relevant. Unfortunately, most women did not agree to collect umbilical cord blood for study.

## 6. Conclusions

In conclusion, the prepregnancy BMI of the examined women influenced newborn birth weight and Ponderal index. Weight gain during pregnancy also influenced the birth weight of newborns. Excessive maternal body weight before or during pregnancy may result in higher birth weight of the newborn, as shown in the study. The adiponectin and leptin concentration was dependent on prepregnancy BMI or pregnancy weight gain. The Ponderal index could be predicted by prepregnancy BMI and leptin plasma concentration in mothers. Maternal overweight or obesity and elevated leptin plasma concentrations can cause metabolic disorders, including excessive fatness in the offspring.

## Figures and Tables

**Table 1:** Weight gain during pregnancy categorized by recommendations by US Institute of Medicine (IOM) among examined women (*n* = 168).

	Weight Gain during Pregnancy		
	Below Standard (*n* = 28)	Standard (*n* = 90)	Above Standard (*n* = 50)
BMI	<12.4 kg	12.4–18.0 kg	>18.0 kg
Underweight (<18.5 kg/m^2^)	4	12	2
	<11.5 kg	11.5–16.0 kg	>16.0 kg
Standard (18.5–24.9 kg/m^2^)	22	52	26
	<7.0 kg	7–11.5 kg	>11.5 kg
Overweight (25.0–29.9 kg/m^2^)	2	20	14
		≤7.0 kg	>7.0 kg
Obese (≥30.0 kg/m^2^)		6	8

**Table 2:** Sociodemographic characteristics of studied women (*n* = 168).

Pregnant Women	
	**Median (Q1; Q2)**
Age (years)	30.5 (28; 34)
Body height (cm)	165.0 (161; 170)
Body weight before pregnancy (kg)	62.0 (54; 71)
Body mass index (BMI) before pregnancy (kg/m^2^)	22.3 (20.4; 25.4)
<18.5 (kg/m^2^) (*n* = 18)	17.5 (17.4; 17.7)
18.5–24.9 (kg/m^2^) (*n* = 100)	21.5 (20.4; 23.3)
25.0–29.9 (kg/m^2^) (*n* =36)	26.7 (25.8; 28.3)
≥30.0 (kg/m^2^) (*n* = 14)	32.7 (31.6; 37.9)
Body weight before delivery (kg)	78.0 (69; 86)
Weight gain during pregnancy (kg)	14 (10; 18)
Below standard (*n* = 28)	7.5 (4; 10)
Standard (*n* = 90)	13.5 (11; 14)
Above standard (*n* = 50)	19.5 (18; 22)
	**(%)**
Place of residence	
City	71
Small town	14
Village	15
Educational level	
Basic education	2
Vocational education	8
Secondary education	24
Higher education	66
Marital status	
Married	76
In a relationship	23
Single	1

**Table 3:** Characteristics of studied neonates (*n* = 168).

Neonates	
	**Median (Q1; Q2)**
Birth length (cm)	54.0 (52; 56)
Birth weight (g)	3350 (3060; 3790)
	**Average ± SD**
Ponderal index (g/cm^3^)	2.18 ± 0.32

**Table 4:** Association between offspring’s anthropometry and prepregnancy body mass index (BMI).

BMI	BMI < 18.5 (kg/m^2^) (*n* = 18)		BMI 18.5–24.9 (kg/m^2^) (*n* = 100)		BMI 25–29.9 (kg/m^2^) (*n* = 36)		BMI ≥ 30 (kg/m^2^) (*n* = 14)		*p*
Birth parameters	Me	(Q1; Q3)	Me	(Q1; Q3)	Me	(Q1; Q3)	Me	(Q1; Q3)	
Birth weight (g) *	3210 ^a^	(3040; 3450)	3300 ^b,c^	(2950; 3600)	3635 ^b^	(3120; 4010)	4005 ^a,c^	(3950; 4190)	*p*^a^ = 0.002 *p* ^b^ = 0.04 *p* ^c^ < 0.001
Birth length (cm) *	55	(52; 57)	54	(51; 56)	54	(52; 56)	55	(53; 58)	*p* > 0.05
	**Average**	**± SD**	**Average**	**± SD**	**Average**	**± SD**	**Average**	**± SD**	***p***
Ponderal index (g/cm^3^) **	1.99 ^d,e^	0.5	2.15 ^f^	0.3	2.28 ^d,f^	0.3	2.45 ^e^	0.4	*p*^d^ = 0.03 *p* ^e^ = 0.04 *p* ^f^ = 0.04

* Kruskal–Wallis test; ** Tukey’s test for different N; ^a^—Statistically significant differences in birth weight of newborns of mothers with BMI indicating underweight and obesity. ^b^—Statistically significant differences in birth weight of newborns of mothers with BMI indicating normal weight and overweight. ^c^—Statistically significant differences in birth weight of newborns of mothers with BMI indicating normal weight and obesity. ^d^—Statistically significant differences in birth Ponderal index of newborns of mothers with BMI indicating underweight and overweight. ^e^—Statistically significant differences in birth Ponderal index of newborns of mothers with BMI indicating underweight and obesity. ^f^—Statistically significant differences in birth Ponderal index of newborns of mothers with BMI indicating normal weight and overweight.

**Table 5:** Association between offspring’s anthropometry at birth and weight gain during pregnancy.

Weight Gain during Pregnancy	Below Standard (*n* = 34)		Standard (*n* = 78)		Above Standard (*n* = 56)		*p*
Birth parameters	Me	(Q1; Q3)	Me	(Q1; Q3)	Me	(Q1; Q3)	
Birth weight (g) *	3255 ^a^	(2875; 3440)	3360	(2960; 3725)	3500 ^a^	(3200; 4000)	*p*^a^ = 0.004
Birth length (cm) *	54	(50.5; 55)	54	(52; 56)	54	(52; 56)	*p* > 0.05
	**Average**	**± SD**	**Average**	**± SD**	**Average**	**± SD**	***p***
Ponderal index (g/cm^3^) **	2.12	0.25	2.14	0.35	2.29	0.3	*p* > 0.05

* Kruskal–Wallis test; ** Tukey’s test for different N; ^a^—Statistically significant differences in birth weight of newborns of mothers with pregnancy weight gain below standard and above standard.

**Table 6:** Association of adiponectin plasma concentration in mothers with age and BMI, weight gain during pregnancy, and offspring’s anthropometry at birth.

		Adiponectin (ng/mL)			
		*n*	Me	(Q1; Q3)	*p*
**Mothers**					
Age *	<30 years	74	4439	(3191; 6503)	*p* = 0.046
	≥30 years	94	5391	(3769; 8280)	
BMI **	<18.5 kg/m^2^	18	5681	(3830; 8360)	*p* > 0.05
	18.5–24.9 kg/m^2^	100	5101	(3342; 7028)	
	24.9–29.9 kg/m^2^	36	4299	2974; 5862)	
	≥ 30 kg/m^2^	14	6445	(5189; 8654)	
Weight gain **	Below standard	34	5895 ^a,b^	(4447; 9460)	*p*_a_ = 0.032 *p*_b_ = 0.031
	Standard	78	4823 ^a^	(3036; 7576)	
	Above standard	56	4561 ^b^	(3314; 6122)	
**Neonates**					
Birth weight **	<2500 g	16	5379	(3791; 10172)	*p* > 0.05
	2500–3999 g	123	5089	(3211; 7826)	
	≥4000 g	29	5436	(3830; 6682)	
Birth length **	<46 cm	10	5379	(4398; 14287)	*p* > 0.05
	46–54 cm	91	5193	(3530; 7833)	
	>54 cm	67	4869	(3192; 6962)	
Ponderal index **	<Standard	51	5700	(2752; 8555)	*p* > 0.05
	<Standard, acceptable	87	5101	(3329; 6794)	
	Standard	30	4515	(4193; 5982)	

* Kruskal–Wallis test; ** Tukey’s test for different N; ^a,b^—statistically significant differences in adiponectin plasma concentration in mothers depending on pregnancy weight gain.

**Table 7:** Association of leptin plasma concentration in mothers with their age and BMI, weight gain during pregnancy, and offspring’s anthropometry at birth.

		Leptin (ng/mL)			
		*n*	Me	(Q1; Q3)	*p*
**Mothers**					
Age *	<30 years	74	29.0	(11.3; 52.1)	*p* > 0.05
	≥30 years	94	25.9	(11.8; 41.5)	
BMI **	<18.5 kg/m^2^	18	9.5 ^a,b^	(5.7; 17.2)	*p*^a^ = 0.013 *p* ^b^ = 0.000 *p* ^c^ = 0.007
	18.5–24.9 kg/m^2^	100	25.8 ^c^	(12.5; 41.5)	
	25–29.9 kg/m^2^	36	35.5 ^a^	21.4; 48.4)	
	≥30 kg/m^2^	14	47.4 ^b,c^	(38.3; 97.1)	
Weight gain **	Below standard	34	13.7 ^d^	(7.9; 37.7)	*p*^d^ = 0.001 *p* ^e^ = 0.003
	Standard	78	23.6 ^e^	(11.5; 40.9)	
	Above standard	56	36.9 ^d,e^	(25.7; 52.2)	
**Neonates**					
Birth weight **	<2500 g	16	29.3	(13.1; 52.8)	*p* > 0.05
	2500–3999 g	123	25.7	(11.3; 42.6)	
	≥4000 g	29	36.8	(23.2; 50.0)	
Birth length **	<46 cm	10	26.8	(6.8; 57.1)	*p* > 0.05
	46–54 cm	91	27.3	(12.3; 41.5)	
	>54 cm	67	26.7	(11.8; 47.7)	
Ponderal index **	<Standard	51	28.9	(10.4; 44.6)	*p* > 0.05
	<Standard, acceptable	87	23.6	(12.0; 39.5)	
	Standard	30	35.9	(25.8; 62.4)	

* Kruskal–Wallis test; ** Tukey’s test for different N; ^a,b,c^—statistically significant differences in leptin plasma concentration in mothers depending on prepregnancy BMI. ^d,e^—statistically significant differences in leptin plasma concentration in mothers depending on pregnancy weight gain.

**Table 8:** Multiple regression analysis considering the adiponectin and leptin.

	Variable	Coefficient	*p*-Value *	R^2^
Adiponectin	BMI (kg/m^2^)	0.093	0.753	0.05
	Body weight gain (kg)	−0.239	0.019	
	Age	0.416	0.186	
Leptin	BMI (kg/m^2^)	2.280	<0.001	0.19
	Body weight gain (kg)	0.387	0.012	
	Age	−0.162	0.732	

**Table 9:** Multiple regression analysis considering the newborn weight, length, and ponderal index.

	Variable	Coefficient	*p*-Value *	R^2^
Newborn weight (g)	BMI (kg/m^2^)	0.449	<0.001	0.14
	Body weight gain (kg)	0.028	0.398	
	Adiponectin (ng/mL)	−0.011	0.672	
Newborn weight (g)	BMI (kg/m^2^)	0.510	<0.001	0.15
	Body weight gain (kg)	0.041	0.215	
	Leptin (ng/mL)	−0.027	0.134	
Newborn length (cm)	Height (cm)	0.208	0.168	0.02
	Adiponectin (ng/mL)	−0.004	0.645	
	Leptin (ng/mL)	0.005	0.422	
Newborn Ponderal index	BMI (kg/m^2^)	0.276	<0.001	0.11
	Body weight gain (kg)	0.025	0.296	
	Adiponectin (ng/mL)	−0.009	0.639	
Newborn Ponderal index	BMI (kg/m^2^)	0.335	<0.001	0.14
	Body weight gain (kg)	0.037	0.116	
	Leptin (ng/mL)	−0.026	0.040	
